# Untargeted plasma and tissue metabolomics in rats with chronic kidney disease given AST-120

**DOI:** 10.1038/srep22526

**Published:** 2016-03-02

**Authors:** Thomas J. Velenosi, Anzel Hennop, David A. Feere, Alvin Tieu, Andrew S. Kucey, Polydoros Kyriacou, Laura E. McCuaig, Stephanie E. Nevison, Michael A. Kerr, Bradley L. Urquhart

**Affiliations:** 1 Department of Physiology and Pharmacology, Schulich School of Medicine and Dentistry, Western University, London, Ontario, Canada; 2 Department of Chemistry, The University of Western Ontario, London, ON, Canada; 3 Lawson Health Research Institute, London, Ontario, Canada; 4 Department of Medicine, Schulich School of Medicine and Dentistry, Western University, London, Ontario, Canada

## Abstract

Chronic kidney disease (CKD) results in the accumulation of metabolic waste products that are normally cleared by the kidney, known as uremia. Many of these waste products are from bacteria metabolites in the gut. Accumulation of uremic toxins in plasma and tissue, as well as the gut-plasma-tissue metabolic axis are important for understanding pathophysiological mechanisms of comorbidities in CKD. In this study, an untargeted metabolomics approach was used to determine uremic toxin accumulation in plasma, liver, heart and kidney tissue in rats with adenine-induced CKD. Rats with CKD were also given AST-120, a spherical carbon adsorbent, to assess metabolic changes in plasma and tissues with the removal of gut-derived uremic toxins. AST-120 decreased >55% of metabolites that were increased in plasma, liver and heart tissue of rats with CKD. CKD was primarily defined by 8 gut-derived uremic toxins, which were significantly increased in plasma and all tissues. These metabolites were derived from aromatic amino acids and soy protein including: indoxyl sulfate, p-cresyl sulfate, hippuric acid, phenyl sulfate, pyrocatechol sulfate, 4-ethylphenyl sulfate, p-cresol glucuronide and equol 7-glucuronide. Our results highlight the importance of diet and gut-derived metabolites in the accumulation of uremic toxins and define the gut-plasma-tissue metabolic axis in CKD.

The National Kidney Foundation defines chronic kidney disease (CKD) as the presence of kidney damage or a progressive decline in renal function lasting for three or more months^1^. The prevalence of CKD is estimated to be upwards of 14% in the United States^2^,^3^,^4^. CKD is divided into 5 stages, which are classified based on glomerular filtration rate. In end stage renal disease, patients require dialysis to sustain life, and the resulting condition is known as the “residual syndrome”^5^. Although biochemical mechanisms of the residual syndrome are not completely understood, uremia is considered a major cause of this condition^5^.

Uremia is the accumulation of solutes that are normally cleared by the kidneys in CKD^6^. At high concentrations, the accumulating solutes are considered uremic toxins as they begin to induce the residual syndrome. Many of these highly concentrated toxins are gut-derived metabolites generated via protein fermentation by bacteria in the colon^7^. Gut-derived aromatic amino acid (AAA) metabolites, such as indoxyl sulfate and p-cresyl sulfate, have been extensively studied in CKD^8^,^9^,^10^,^11^. Indoxyl sulfate and p-cresyl sulfate play a significant role in cardiovascular disease, which is the leading cause of death in patients with CKD accounting for 45% of mortalities^12^,^13^,^14^. Indoxyl sulfate concentration is correlated with aortic calcification and vascular stiffness in patients with CKD^15^. High plasma levels of unbound p-cresyl sulfate increases the risk of cardiovascular and all-cause mortality in hemodialysis patients^16^.

AST-120 is a spherical carbon adsorbent that has been used in Japanese CKD patients since 1991 to remove the precursors of uremic toxins produced in the gut. This compound is formulated to bind small molecules (100 to 10,000 Da) while larger molecules such as digestive enzymes and hormones are left unbound^18^. Adsorption of gut-derived uremic precursors to AST-120 decreases circulating levels of these toxins. In clinical trials, AST-120 has been shown to mitigate symptoms of uremia in CKD^18^,^19^.

A number of studies have quantified individual uremic toxin levels in patients with CKD. In 2003, the European Uremic Toxin Work Group compiled mean and median serum or blood levels of 90 uremic toxins using meta-analysis^11^. Subsequent updates were published in 2007 and 2012, adding 14 and 56 uremic toxins, respectively^10^,^20^. This is an effective method for determining mean patient uremic toxin levels; however, the uremic toxins reported are a result of targeted analysis, which can limit the identification of novel toxins. Recent advances in mass spectrometry allow for identification of novel uremic toxins using an untargeted metabolomics approach.

Metabolomics is the study of all metabolites generated in a biological system. Thousands of metabolites can be detected using untargeted metabolomics without prior knowledge of their composition^21^. In the setting of CKD, metabolomics can provide uremic signatures directly associated with decreased renal function^22^. This information can be used to determine mechanisms of kidney disease initiation and progression. Metabolomics has been applied to diabetic nephropathy, polycystic kidney disease, acute kidney injury and renal cell carcinoma in patients^23^. Recently, clinical and animal studies have begun to identify plasma uremic toxins using untargeted metabolomics^24^,^25^,^26^. These metabolites have been identified using both NMR and LC-MS techniques. Advances in LC-MS using ultra performance liquid chromatography (UPLC) coupled with quadrupole time-of-flight mass spectrometry (UPLC-QToF/MS) allows for high throughput and highly sensitive metabolomics analysis. The advent of UPLC has resulted in increased chromatographic resolution and improved compound separation^27^. Current QToF/MS techniques allow alternating scans of low and high collision energy for simultaneous acquisition of precursor and fragment ions in a single run, thereby improving workflow^28^.

Plasma uremic toxin levels have been correlated with CKD complications, most notably are the cardiovascular effects including endothelial dysfunction and heart failure^13^,^29^. Metabolic changes in the kidney are known to occur as a result of kidney damage, and the accumulation of uremic toxins has been shown to further kidney injury^30^,^31^. However, effects of accumulating toxins on metabolic changes in other major organs such as the heart and liver have not been assessed. Therefore, the purpose of this study was to determine the metabolic changes in plasma, liver, heart and kidney tissue as well as the metabolic axis between these matrices in rats with CKD. We also assessed the plasma and tissue metabolic effects of removing gut-derived uremic toxins using AST-120 to further understand the gut contribution to the uremic condition in both plasma and tissues in CKD.

## Results

### Validation of Experimental Model

The extent of CKD was assessed by plasma creatinine and urea levels (Table 1). Creatinine levels were 8.0-fold and 5.8-fold higher in CKD and CKD+AST-120 groups, respectively, compared to controls (p < 0.05). CKD and CKD+AST-120 rats had a 6.8 and 5.1-fold increase in plasma urea levels compared to controls. There were no significant differences in weights between each group.

### Principle Component Analysis (PCA) and the Removal of Metabolites by AST-120 in Biological Matrices

**Figure 1:**
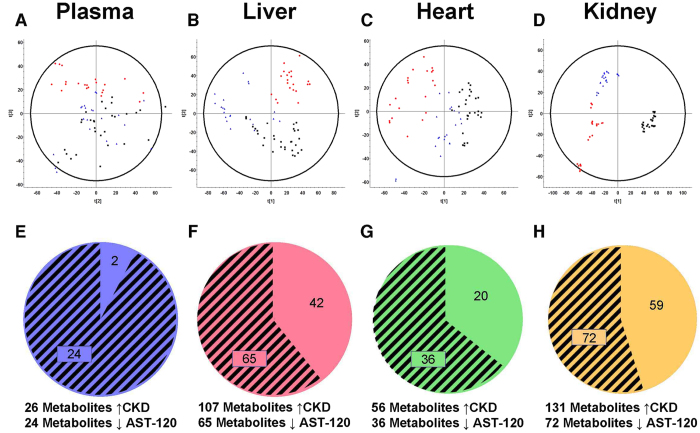
Principle component analysis and metabolites increased in CKD and decreased by AST-120 in negative ESI mode. Control (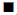), CKD (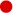) and CKD+AST-120 (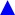) negative ESI mode principle component analysis of plasma (**A**), liver (**B**), heart (**C**) and kidney (**D**) tissue in rats. Triplicate injections are shown. Metabolites significantly increased in CKD compared to control (represented as whole pie) and significantly decreased in CKD+AST-120 compared to CKD (represented as shaded pie) for plasma (**E**), liver (**F**), heart (**G**) and kidney (**H**) tissue. Metabolites were considered significantly altered using one-way ANOVA (p < 0.01, q < 0.001, and maximum intensity > 1500) and Tukey’s post-hoc test.

**Figure 2:**
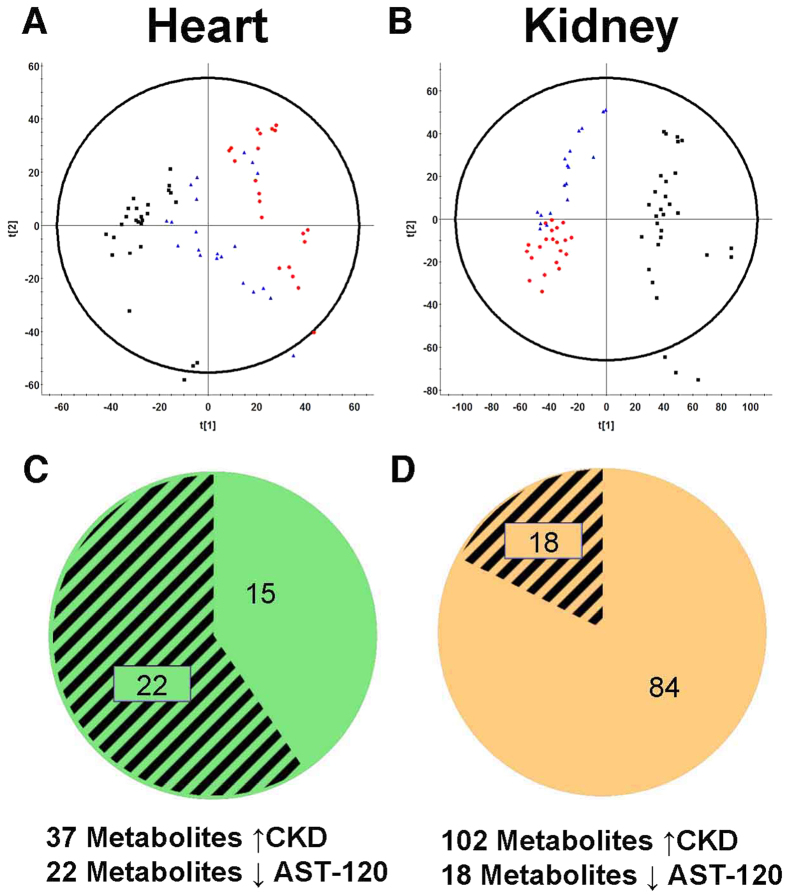
Principle component analysis and metabolites increased in CKD and decreased by AST-120 in positive ESI mode. Control (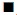), CKD (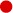) and CKD+AST-120 (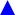) positive ESI mode principle component analysis of heart (**A**) and kidney (**B**) tissue in rats. Metabolites significantly increased in CKD compared to control (represented as whole pie) and significantly decreased in CKD+AST-120 compared to CKD (represented as shaded pie) for heart (**C**), kidney (**D**). Triplicate injections are shown for principle component analysis. Metabolites were considered significantly altered using one-way ANOVA (p < 0.01, q < 0.001, and maximum intensity > 1500) and Tukey’s post-hoc test.

To determine the metabolic changes in CKD with and without the administration of AST-120, samples were analyzed by ultra performance liquid chromatography with quadrupole time-of-flight mass spectrometry (UPLC-QToF/MS) in positive and negative electrospray ionization (ESI) modes. All samples, including pooled samples, were evaluated by multivariate analysis. Representative UPLC-QToF/MS chromatograms as well as an overlay of extracted ion chromatograms for all metabolites identified in negative and positive ESI modes are demonstrated by effective chromatographic resolution (Supplementary Figure S1). Pooled sample injections demonstrated reproducibility and were clustered in the center of principle component analysis plots (Supplementary Figure S2 and S3). The first component in plasma negative mode PCA did not show clustering of experimental groups. When using second and third components, CKD rat plasma samples appeared as a separate cluster compared to control and CKD+AST-120 groups (Fig. 1A). The control and CKD+AST-120 samples were also grouped together in this analysis. The majority of metabolites were increased in CKD relative to control and CKD+AST-120 groups. Using XCMS Online multi-group experiment, 26 metabolites were significantly increased in CKD plasma samples compared to control. Of those 26 metabolites, 24 were significantly reduced by the administration of AST-120 to rats with CKD (Fig. 1E). The PCA for liver metabolites in negative mode also demonstrated clustering of CKD samples; however, CKD+AST-120 showed some separation from control samples. Further analysis revealed that AST-120 removed 60.7% of metabolites that were significantly increased in CKD rat livers (Fig. 1F). Positive ESI did not show marked differences between groups for plasma and liver samples. The metabolic profile in heart tissue was distinctly different between control and CKD rats with complete separation of samples in the PCA for both negative and positive ESI modes (Figs 1C and 2A). CKD+AST-120 rat samples were clustered between control and CKD groups. AST-120 significantly decreased 58 of 93 metabolites in heart tissue that were increased in CKD (Figs 1G and 2C). Metabolites between control, CKD and CKD+AST-120 kidney tissue were mutually exclusive in the PCA for negative ESI mode showing the greatest separation between all matrices analyzed (Fig. 1D). In positive ESI mode, control and CKD components were well separated with slight overlap between CKD and CKD+AST-120 groups. Kidney tissue had the largest number of significantly increased metabolites in CKD compared to control. However, AST-120 reduced less than 40% (90 of 233) of metabolites that were significantly increased in CKD compared to control kidneys (Figs 1H and 2D).

### Orthogonal Partial Least Squares Discriminant Analysis (OPLS-DA) of Control and CKD Biological Matrices

**Figure 3:**
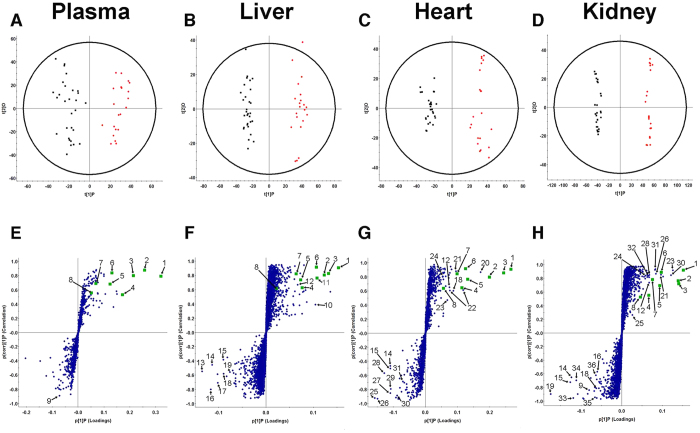
Orthogonal partial least squares discriminant analysis (OPLS-DA) and S-plots of control and CKD biological matrices in negative ESI mode. Control (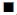) and CKD (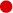) negative ESI mode OPLS-DA of plasma (**A**) (R^2^(Y) = 0.92, Q^2^(Y) = 0.75), liver (**B**) (R^2^(Y) = 0.98, Q^2^(Y) = 0.87), heart (**C**) (R^2^(Y) = 0.97, Q^2^(Y) = 0.89), and kidney (**D**) (R^2^(Y) = 1.00, Q^2^(Y) = 0.95) tissue in rats. Triplicate injections are shown. S-plots showing ions that define the CKD group in the upper right quadrant and ions that define control group in the lower left quadrant for plasma (**E**), liver (**F**), heart (**G**) and kidney (**H**) tissue. Ions with the greatest contribution in separating control and CKD groups are labeled and defined in Table 2 and Supplementary Table S5. Gut-derived metabolites that are increased in CKD and shared by all tissues are labeled 1 to 8 (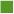).

**Figure 4:**
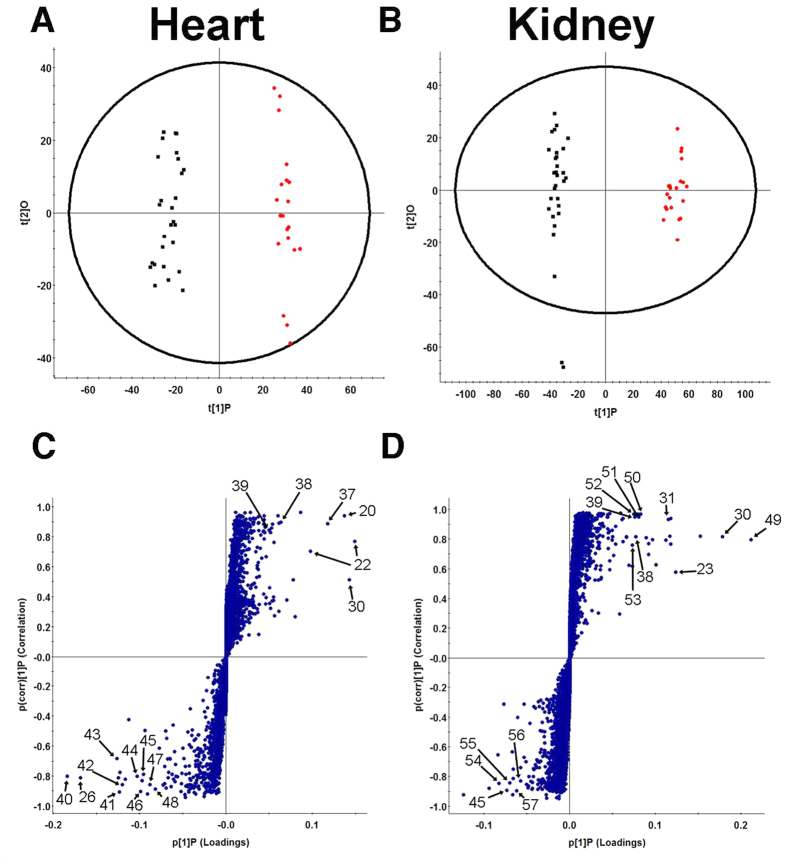
Orthogonal partial least squares discriminant analysis (OPLS-DA) and S-plots of control and CKD biological matrices in positive ESI mode. Control (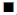) and CKD (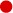) positive ESI mode OPLS-DA of heart (**A**) (R^2^(Y) = 0.98, Q^2^(Y) = 0.86), and kidney (**B**) (R^2^(Y) = 0.99, Q^2^(Y) = 0.92) tissue in rats. Triplicate injections are shown. S-plots showing ions that define the CKD group in the upper right quadrant and ions that define control group in the lower left quadrant for heart (**C**) and kidney (**D**) tissue. Ions with the greatest contribution in separating control and CKD groups are labeled and defined in Supplementary Table S5.

The metabolites responsible for the differences between control and CKD rats were assessed by OPLS-DA in plasma, liver, heart and kidney. Plasma metabolites in control and CKD samples were well described by OPLS-DA (R^2^(Y) = 0.92) with high predictive ability (Q^2^(Y) = 0.75). The majority of metabolites in plasma samples were increased in CKD relative to control. Many of these metabolites were AAA derivatives including phenyl sulfate, indoxyl sulfate, p-cresyl sulfate, 4-ethylphenyl sulfate, hippuric acid and p-cresol glucuronide (Fig. 3E and Table 2). Tissue samples from rats with CKD also had high levels AAA derivatives. Few metabolites were significantly higher in control plasma samples compared to CKD. However, tryptophan and many fatty acids including palmitic acid, oleic acid, linoleic acid, arachidonic acid, eicosapentaenoic acid (EPA) and docosahexaenoic acid (DHA) were present at higher levels in control livers. CKD rat livers were described by increases in equol sulfate and taurocholic acid compared to control livers along with a similar AAA derivative profile as CKD plasma in OPLS-DA (Fig. 3F, R^2^(Y) = 0.98, Q^2^(Y) = 0.87). In heart tissue samples run in negative ESI mode, metabolites in the control group were defined by lysophosphatidylcholines and lysophosphoethanolamines (Fig. 3G, R^2^(Y) = 0.97, Q^2^(Y) = 0.89). Heart metabolites that were increased in CKD included AAA derivatives as well as pantothenic acid and glutathione, which also appeared in positive ESI mode (Fig. 3G and 4C). Positive ESI OPLS-DA demonstrated increased levels of L-carnitine and L-carnitine derivatives in heart tissue of control rats compared to CKD. Rat kidneys had the greatest separation in negative and positive ESI mode OPLS-DA plots, and showed the best fit and predictive ability compared to all other biological matrices (Fig. 3D, R^2^(Y) = 1.0, Q^2^(Y) = 0.95, Fig. 4B, R^2^(Y) = 0.99, Q^2^(Y) = 0.92). CKD kidneys had higher levels of AAA derivatives and adrenic acid. Both positive and negative ESI mode demonstrated increased levels of lysophosphatidylcholines and lysophosphoethanolamines in CKD. Control kidneys had higher levels of similar fatty acids seen in control livers including linoleic acid and arachidonic acid. DHA glychocholic acid, L-carnitine and L-carnitine derivatives were also increased in control kidneys compared to CKD.

### Common Significant Metabolites

**Figure 5:**
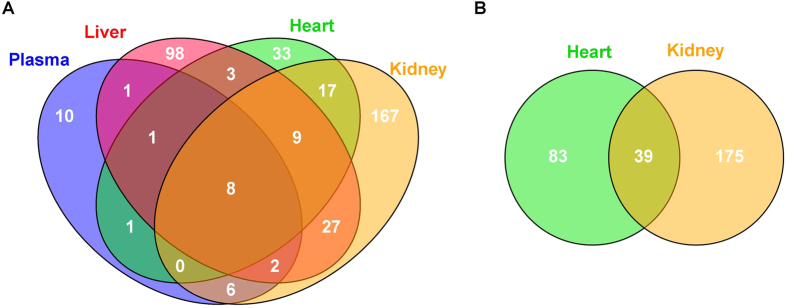
Meta-analysis of significantly different metabolites between control and CKD in biological matrices. A second-order analysis of significantly different metabolites in negative ESI mode for plasma, heart, liver and kidney tissue (**A**) and positive ESI mode for heart and kidney tissue (**B**). Highly significant metabolites (p < 0.01, q < 0.001, and maximum intensity > 1500) were used for second-order analysis. Second-order analysis parameters included fold-changes ≥ 1.5 and p < 0.05 for Tukey’s post-hoc test. Common metabolites between biological matrices were aligned using a m/z = 0.01 and a retention time = 20 s tolerance. Information about the 8 common metabolites between all biological matrices in negative ESI mode is listed in Table 2.

**Figure 6:**
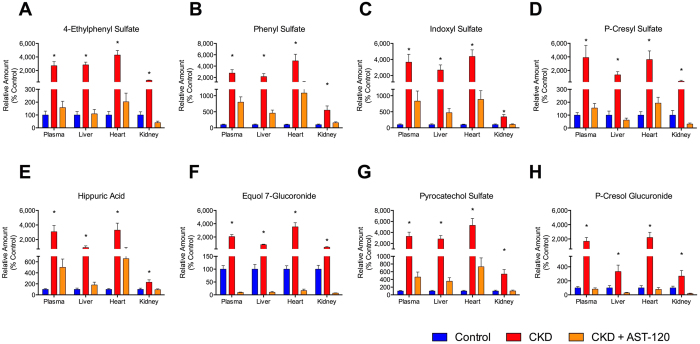
Common significantly different metabolites found in all biological matrices. Plasma, liver, heart and kidney levels of the 8 gut-derived metabolites found to be significantly different in Fig. 5A: 4-ethylphenyl sulfate (**A**), phenyl sulfate (**B**), indoxyl sulfate (**C**), p-cresyl sulfate (**D**), hippuric acid (**E**), equol 7-glucuronide (**F**), pyrocatechol suflate (**G**) and p-cresyl glucuronide (**H**). Controls were arbitrarily defined as 100%. Results are means ± SEM; n = 10 (control) and n = 7 (CKD and CKD+AST-120). *p < 0.05 vs control and CKD+AST-120.

A second order analysis in metaXCMS was used to determine common metabolic differences in plasma, liver, heart and kidney tissue between control and CKD rats. All tissues shared 8 common metabolites, which were significantly increased in CKD compared to control rats (Fig. 5A). All 8 metabolites were gut derived uremic toxins and included the AAA metabolites: phenyl sulfate, indoxyl sulfate, p-cresyl sulfate, hippuric acid, pyrocatechol sulfate and p-cresol glucuronide as well as 4-ethylphenyl sulfate and equol 7-glucuronide (Fig. 6, Table 2). These 8 metabolites were significantly decreased in CKD+AST-120 rats compared to controls. Plasma levels of these metabolites also showed a significant correlation with liver, heart and kidney levels (Table 2, Supplementary Figure S4). In positive ESI mode, L-carnitine and many of the L-carnitine derivatives found to be significantly different between control and CKD rats in pairwise comparisons were shared between heart and kidney tissue (Fig. 5B). Metabolite pathway analysis demonstrated significant metabolic disturbances in a number of pathways involved with lipid, bile acid and amino acid metabolism (see Supplementary Table S6 for details).

## Discussion

In this study, rat plasma, liver, heart and kidney metabolic signatures were evaluated in CKD with and without the administration of AST-120. Rat plasma metabolic changes in CKD have been assessed in previous studies with the use of AST-120^32^,^33^. Renal metabolic profiles in negative ESI mode have also been evaluated in rats with CKD^34^; however, this study is the first to assess tissue metabolic signatures in heart, liver and kidney including the metabolic axis between plasma and tissues. We demonstrate that the uremic condition causes a number of metabolic changes in both plasma and tissues in CKD and that many of these can be mitigated with AST-120.

The accumulation of uremic toxins in plasma and their contribution to the residual syndrome can be deleterious to patients with CKD^5^. Non-invasive plasma sampling is crucial for evaluating the uremic condition in patients; however, to further our understanding of the metabolic interaction between plasma and tissues in CKD, tissue metabolic signatures must be directly assessed. Interestingly, less than 14% of metabolites that were significantly different between control and CKD rats in liver, heart and kidney tissues were also significantly different in plasma. Therefore, tissue metabolic signatures of CKD were weakly represented in plasma samples.

Rats with CKD had reduced kidney function that was not significantly affected by AST-120. The majority of plasma metabolites increased in CKD were reduced by administration of AST-120, suggesting that a number of these metabolites are derived from the gut or produced from the effects of gut-derived uremic toxins on *in vivo* metabolic processes. Although kidney function was not altered by AST-120, more than 50% of metabolites were reduced in both liver and heart tissue metabolic profiles with AST-120 administration to rats with CKD. Therefore, a number of tissue metabolic processes are affected by gut-derived metabolites.

The 8 metabolites that were significantly increased in all biological matrices were also significantly reduced by AST-120. P-cresyl sulfate, p-cresyl glucuronide, indoxyl sulfate, hippuric acid and phenyl sulfate are known gut-derived uremic toxins^7^. Gut precursors of p-cresyl sulfate, indoxyl sulfate, p-cresyl glucuronide and phenyl sulfate undergo sulfation or glucuronidation, which occurs through phase II drug metabolizing enzymes^33^,^35^. The major site of phase II drug metabolism is in the liver and therefore, hepatic metabolism of gut precursors plays a major role in the accumulation of these toxins. Gut-derived toxins can also affect hepatic drug metabolizing enzymes. Cytochrome P450 3A4 metabolizes approximately 40% of drugs on the market and indoxyl sulfate levels have been correlated with decreased levels of the CYP3A4 endogenous metabolite, 4β-hydroxycholesterol^36^,^37^. Once in the blood stream, these uremic toxins are also highly protein bound and both p-cresyl sulfate and indoxyl sulfate renal clearance is mediated by organic anion transporters (OAT) 1 and 3 in the kidney^38^,^39^. Only the free fraction of these uremic toxins can exhibit biological effects or undergo transport into cells. In OAT1 knockout mice, indoxyl sulfate, p-cresyl sulfate, phenyl sulfate and pantothenic acid accumulate in plasma^38^. Therefore, increased levels of these toxins in both plasma and tissues are likely a result of decreased OAT1 function in the kidneys of rats with CKD.

In this study, we demonstrate for the first time that both p-cresyl sulfate and indoxyl sulfate accumulate in heart tissue and can be mitigated by AST-120 *in vivo*. P-cresyl sulfate is a gut-derived bacterial metabolite produced from tyrosine. In a prospective clinical study, free p-cresyl sulfate levels predicted cardiovascular mortality and all-cause mortality in dialysis patients^16^. The effect of p-cresyl sulfate on cardiac toxicity and dysfunction were recently published in rats with CKD suggesting NADPH oxidase activation and ROS as possible mechanisms in p-cresyl sulfate mediated cardiac apoptosis^40^. Indoxyl sulfate is formed from tryptophan and has been shown to cause pro-fibrotic, pro-inflammatory and pro-hypertrophic affects through p38 MAPK, p42/44 MAPK, and NF-κB pathways in both rat cardiac myocytes and fibroblasts *in vitro*^41^. In patients, indoxyl sulfate has been associated with the first heart failure event, and removal of indoxyl sulfate by AST-120 has been shown to improve cardiac function^13^,^29^. Prevention of cardiovascular disease in CKD is essential as these patients are more likely to die of cardiovascular disease than progress to requiring dialysis^42^. P-cresyl sulfate and indoxyl sulfate are increased 361-fold and 438-fold, respectively, in rat CKD heart tissue compared to control (Fig. 6A,B). Therefore, accumulation of these uremic toxins in the heart may provide evidence for potential mechanisms of cardiovascular disease development in CKD.

Pyrocatechol sulfate is a dietary phenolic metabolite that has not been previously described as a uremic toxin^43^. Equol 7-glucoronide and 4-ethyl phenyl sulfate are also produced from gut bacteria but are specifically derived from soy protein. Standard rat chow includes soy as the main source of protein and the diet used in this study contained daidzein and genistein aglycone equivalents between 350 and 650 mg/kg, which are metabolized by gut bacteria into precursors of equol 7-glucuronide and 4-ethylphenyl sulfate, respectively^44^,^45^. These uremic toxins have not been identified in patients with CKD. Intestinal bacteria produce equol in 20–30% of Western populations and 40–60% of Asian populations^44^,^46^. Consequently, equol may only accumulate in patients who both consume soy protein and have the gut bacteria to convert daidzein to equol. Although all patients with CKD will have high AAA derived uremic toxins levels, accumulation of equol 7-glucuronide and 4-ethyl phenyl sulfate demonstrate the importance of diet on the production of uremic toxins in CKD. The 8 common metabolites identified had the highest variable importance in projection (VIP) values defining CKD in plasma and tissue S-plots (Fig. 3 and Table 2). Therefore, gut-derived uremic toxins provide the most definitive metabolic signatures of both plasma and tissue in CKD. Tissue accumulation of these gut-derived uremic toxins in patients is unknown; however, in this study, we demonstrate that plasma levels of the 8 gut-derived metabolites were highly correlated with tissue levels (Table 2). This suggests that the non-invasive measurement of these 8 gut-derived uremic toxins in plasma may be used as a surrogate for tissue levels in CKD.

In control animals, higher levels of tryptophan were observed, which has also been demonstrated previously^47^. Indoxyl sulfate is formed from tryptophan and elevated levels of indoxyl sulfate have been shown to reduce tryptophan binding to serum albumin resulting in increased levels of free tryptophan^48^. Increasing free tryptophan levels subsequently increases tryptophan availability for uptake into tissues for metabolism, thereby reducing plasma levels^49^. This effect is mitigated in patients with the administration of AST-120^50^. In liver tissue, free fatty acids were decreased in animals with CKD. These included palmitic acid, oleic acid, linoleic acid, arachidonic acid, EPA and DHA (Fig. 3 and Supplementary Table S5). All of these fatty acids are components in the rat diet. Metabolite pathway analysis of these fatty acids demonstrates their involvement in a number of pathways; however, for the majority of fatty acid pathways, only one metabolite was matched. Palmitic acid was matched in the fatty acid biosynthesis, fatty acid metabolism and fatty acid elongation in the mitochondria pathways. AST-120 recovered hepatic levels of free palmitic acid suggesting that gut-derived uremic toxins may be involved in perturbing these pathways. Indeed, a previous study identified increased palmitic acid levels in feces of rats with adenine-induced CKD^31^. Therefore, future targeted studies are necessary to determine the contribution of reduced dietary absorption and metabolic pathway disturbances in the liver resulting in decreased hepatic free fatty acids levels in CKD. The depletion of both EPA and DHA in kidney tissue of rats with CKD is demonstrated in this study and others^30^,^34^. Although plasma levels of these fatty acids were not detected, patients on dialysis have lower plasma levels of EPA and DHA, which are unaltered in non-dialysis CKD patients^51^. A recent clinical study supplementing CKD patients with omega-3 fatty acids showed increases in specialized pro-resolving lipid mediators, which can inhibit pro-inflammatory cytokine production and potentially decrease low grade inflammation in CKD^52^. AST-120 recovered levels of a number of free fatty acids in animals with CKD (see Supplementary Table S5). However, the mechanisms of AST-120 ameliorating the reduction in free fatty acid levels for rats with CKD are unknown. Therefore, targeted metabolomics studies assessing compounds in these metabolic pathways are necessary to understand the mechanisms resulting in metabolic disturbances in CKD.

Fatty acids are an important energy source for cardiac tissue, which can be utilized via β-oxidation^53^. It is estimated that 50–70% of ATP generated in the adult heart is a result of β-oxidation^54^. Although saturated fatty acids such as palmitic acid undergo β-oxidation, the majority of β-oxidation occurs through unsaturated fatty acids including oleic acid and arachidonic acid^54^. L-carnitine mediated transport of long chain fatty acids into the mitochondria is the rate-limiting step in fatty acid oxidation^55^. In this study, changes in heart fatty acid levels were not consistent; however, we demonstrate decreased levels of L-carnitine and L-carnitine derivatives in heart tissue from rats with CKD. Cardiac tissue cannot synthesize L-carnitine, which must be obtained through plasma from dietary sources or production in the liver and kidney. L-carnitine is cleared through the dialysis membrane resulting in decreased levels for dialysis patients; however, levels in CKD patients are not correlated with kidney function^56^. In humans, 75% of L-carnitine is obtained from the diet^55^,^57^. The standard rat diet used in this study was not supplemented with L-carnitine. As a result, it is possible that synthesis of L-carnitine is decreased in rats with CKD; however, this effect is not seen in patients due to substantial dietary L-carnitine consumption. AST-120 recovered levels of L-carnitine in CKD rats and therefore gut-derived uremic toxins may affect the synthesis of L-carnitine (see Supplementary Table S5).

In summary, gut-derived metabolic signatures most prominently defined plasma, liver, heart and kidney tissues in rats with CKD. The majority of these compounds were AAA metabolites. Increased levels of soy protein metabolites 4-ethyl phenyl sulfate and equol-glucuronide, which have not been described in patients with CKD, emphasize the potential for dietary effects on individual uremic toxin profiles. This study also highlights the gut-plasma-tissue metabolic axis, directly demonstrating the accumulation of gut-derived uremic toxins in multiple major organs of rats with CKD.

## Materials and Methods

### Chemicals and Reagents

Harlan 8640 Teklad 22/5 rodent diets were used for both control and 0.7% adenine supplemented diets (Madison, WI). Indoxyl sulfate was obtained from gold biotechnology (Olivette, MO). P-cresyl glucuronide and equol 7-glucuronide were purchased from Toronto Research Chemicals (Toronto, ON, Canada) and β-glucuronidase from *Helix pomatia* (G-7051), pyrocatechol, p-cresol and 4-ethylphenol were obtained from Sigma-Aldrich (St. Louis, MO). Flurazepam was purchased from Cerilliant (Round Rock, TX). Isatin was purchased from Alfa Aesar (Ward Hill, MA). P-cresyl sulfate, phenyl sulfate and 4-ethyl phenyl sulfate were synthesized as previously described^58^. AST-120 was a kind gift from the Kureha Corporation (Tokyo, Japan).

### Animal Models

A total of 30 male Wistar rats weighing 150 g were obtained from Charles River Laboratories, Inc. (Wilmington, MA) for this study. Animal care and experimental protocols and procedures were approved by the Western University Animal Care Committee and conducted in accordance with the Canadian Council on Animal Care. Rats initially weighed 150 g and after 5 days of acclimatization were divided into two groups: control (n = 10) and CKD (n = 20). The CKD group received 0.7% adenine supplemented into rat chow ad libitum for 5 weeks to induce kidney disease. Food was weighed daily for the CKD group and pair-fed as standard chow to control animals. After 5 weeks, the CKD group was equally divided and the diet was changed to standard chow (CKD, n = 10) or standard rat chow supplemented with 8% AST-120 (CKD+AST-120, n = 10) for an additional 3 weeks. All animals were weighed daily throughout the study. Three rats from the CKD and CKD+AST-120 groups experienced significant weight loss and were euthanized prior to the end of the study. At the conclusion of the study, control (n = 10), CKD (n = 7) and CKD+AST-120 (n = 7) rats were sacrificed and blood was obtained in heparinized tubes to acquire plasma. Liver, heart and left kidneys were removed, snap-frozen in liquid nitrogen and stored at −80 °C. Plasma creatinine and urea were determined by the London Laboratory Services Group (London, ON, Canada) using standard methods.

### Sample Preparation and Batch Setup

Plasma samples were thawed and protein was precipitated with the addition of ice-cold acetonitrile (3:1, acetonitrile:plasma). Liver, heart and kidney tissue (100 mg) were homogenized in 250 μL of ice-cold acetonitrile. All samples were placed on ice for 20 minutes and centrifuged at 14,000 g for 5 minutes. The supernatant from tissue samples was diluted to 80% acetonitrile. Quality control samples were generated for each matrix by creating a pooled sample. Acetonitrile used for protein precipitation contained isatin (5 μg/mL) and flurazepam (25 ng/mL) as internal standards. Samples from individual rats were transferred to vials and 1 μL was injected in triplicate for each vial. Injections were randomized to reduce error associated with instrument drift and quality control pool samples were run every 6 injections. All samples were run in a single batch for each biological matrix.

### Chromatographic Separation and Mass Spectrometry

Metabolites were separated using a Waters ACQUITY UPLC HSS T3 column (1.8 μm particle size, 100 mm × 2.1 mm). The column was maintained at 45 °C in a Waters ACQUITY UPLC I-Class system (Waters, Milford, MA). The mobile phase flow was set to 0.45 ml/min and consisted of water (A) and acetonitrile (B), both containing 0.1% formic acid. The UPLC conditions were as follows: 0–2 mins, 1–60% B; 2–6 mins 60–85% B; 6–8 mins 85–99% B; 8–10 mins 99–1% B. Mass spectrometry was performed using a Waters Xevo^TM^ G2S-QTof/MS. Metabolites were measured separately in positive and negative ESI modes. Capillary voltage and cone voltage were set at 2 kV and 40 V, respectively. The source temperature was 150 °C. The desolvation gas flow was set to 1200 L/h at 600 °C and the cone gas flow was 50 L/h. The data was acquired in centroid mode using an MS^E^ method, which allows for both MS and MS/MS fragmentation in a single run. Functions 1 and 2 of the MS^E^ method acquired data with a 0.1 s scan time in the range of 50–1200 m/z, which was calibrated using sodium formate clusters prior to running samples. Collision energy was set to 0 V and ramped from 15–50V for functions 1 and 2, respectively. Function 3 acquired lockspray to ensure mass accuracy. Leucine-enkephalin (500 ng/mL) was used as the lockmass set at a flow rate of 10 μL/min, measured every 10 s and averaged over 3 scans.

### Data Analysis

#### Mutlivariate Data Analysis

Mutlivariate analysis of LC-MS data was achieved by Waters Markerlynx with EZinfo 2.0 (Umetrics, Umeå, Sweden) software packages. Peak intensities were normalized to total marker intensity in Markerlynx and subsequently transferred to EZinfo. Pareto scaling was used to dampen the selection of features with the highest variance. EZinfo was used for principle component analysis (PCA) of plasma, liver, heart and kidney, as well as orthogonal partial least squares discriminant analysis (OPLS-DA) between control and CKD rats for each matrix.

#### Univariate Data Analysis

XCMS Online^59^ (https://xcmsonline.scripps.edu) was used for univariate data analysis. Raw data files were initially converted to mzData files and the lockmass was removed using massWolf 4.3.1 and the chemhelper package in R version 3.2.0. Function 1 MS data was uploaded to XCMS Online and the data was processed as a multi-group experiment using the default UPLC with G2S MS parameters. Briefly, the centWave method was used for feature detection (m/z tolerance = 15 ppm, min peak width = 2 s, max peak width = 25 s). The Obiwarp method was used for retention time correction (profStep = 0.5) and chromatograms were aligned using the following parameters: mzwid = 0.1, minfrac = 0.5, bw = 2. Metabolite features were statistically analyzed using one-way ANOVA with Tukey’s post-hoc test. Isotopes and adducts were annotated using CAMERA (m/z absolute error = 0.015, ppm = 5) and arranged into feature groups. Features for each biological matrix were filtered for isotopes. The feature with the maximum intensity in each feature group and present in at least 80% of pooled sample injections was chosen for statistical analysis. Highly significant metabolites were selected to assess differences between biological matrices in control, CKD, and CKD+AST-120 rats (p < 0.01, q < 0.001, maximum intensity > 1500).

Pairwise metabolite differences between control and CKD rats that were common across all biological matrices were determined using metaXCMS^60^. Highly significant metabolites for each tissue were aligned using a 20 second retention time tolerance and a 0.01 m/z tolerance.

### Metabolite Identification

Metabolites that were considered highly significant between control and CKD rats from multivariate and univariate analysis were searched in METLIN and Human Metabolome Database (HMDB) metabolomics databases. Fragmentation patterns for each metabolite were compared to putative database compound fragmentation using MassFragment®. Compound standards were purchased or synthesized, and retention time and fragmentation pattern were compared to confirm the metabolite identity. Pyrocatechol sulfate was identified by incubation of plasma with β-glucuronidase from *Helix pomatia*, which has ≥10,000 units/g sulfatase activity. The sulfatase product fragmentation pattern was confirmed as pyrocatechol to a purchased standard.

### Metabolite Pathway Analysis

Pathway analysis of identified metabolites was performed in MetaboAnalyst 3.0^61^,^62^. GlobalTest was used for pathway enrichment analysis and relative-betweeness centrality was selected for pathway topology analysis.

## Additional Information

**How to cite this article**: Velenosi, T. J. *et al*. Untargeted plasma and tissue metabolomics in rats with chronic kidney disease given AST-120. *Sci. Rep.* **6**, 22526; doi: 10.1038/srep22526 (2016).

## Supplementary Material

Supplementary Information

## Figures and Tables

**Table 1:** Physical and biochemical characteristics of control, CKD and CKD+AST-120 rats.

	Control (n = 10)	CKD (n = 7)	CKD+AST-120 (n = 7)
Body Weight (g)	277 ± 12	297 ± 18	258 ± 23
Serum Creatinine (μM)	23 ± 1	181 ± 22*	130 ± 17*
Serum Urea (mM)	6.6 ± 0.5	44.9 ± 5.6*	33.6 ± 5.8*

Data are presented as means ± SEM.

^*^p < 0.05 compared to control.

**Table 2:** Summary of metabolites altered in plasma, liver, heart and kidney tissue in rats with CKD compared to control and CKD+AST-120.

Ion	*t*_R_ (min)	Mass (m/z)	Empirical Formula	Mass Error (ppm)	Identity	Tissue	Change compared to Control				Change compared to CKD+AST-120			Metabolite ID Level	Correlation with Plasma Levels (R^2^)
								S-plot VIP	Fold	P-value		Fold	P-value		
1	2.28	201.0223	C8H10O4S[H-]	0.5	4-Phenylethyl Sulfate	Plasma	↑	12.26	27.84	2.08E-10	↑	17.33	2.08E-10	1	
						Liver	↑	15.01	35.88	4.89E-10	↑	23.42	4.89E-10		0.74
						Heart	↑	16.96	44.31	4.89E-10	↑	19.40	4.89E-10		0.93
						Kidney	↑	14.17	5.81	4.89E-10	↑	12.61	4.89E-10		0.58
2	1.63	172.9908	C6H5O4S[H-]	0.6	Phenyl Sulfate	Plasma	↑	9.55	29.41	2.08E-10	↑	3.43	2.12E-10	1	
						Liver	↑	12.16	28.02	4.89E-10	↑	4.25	4.89E-10		0.91
						Heart	↑	13.26	39.22	4.89E-10	↑	3.12	2.06E-09		0.86
						Kidney	↑	11.06	5.46	4.89E-10	↑	3.35	4.91E-10		0.81
3	1.71	212.0018	C8H6NO4S[H-]	0	Indoxyl Sulfate	Plasma	↑	8.12	39.07	2.08E-10	↑	4.41	1.20E-09	1	
						Liver	↑	13.12	31.58	4.89E-10	↑	5.12	4.89E-10		0.78
						Heart	↑	16.24	42.60	4.89E-10	↑	4.38	4.89E-10		0.76
						Kidney	↑	11.22	3.62	4.89E-10	↑	3.14	4.91E-10		0.69
4	1.93	187.0065	C7H7O4S[H-]	0	P-Cresyl Sulfate	Plasma	↑	7.09	40.84	1.43E-07	↑	26.36	1.56E-06	1	
						Liver	↑	7.46	19.01	4.94E-10	↑	19.62	5.83E-10		0.82^a^
						Heart	↑	7.87	40.03	4.90E-10	↑	15.58	5.53E-10		0.84^a^
						Kidney	↑	5.31	4.17	4.16E-08	↑	12.29	1.90E-09		0.68^a^
5	1.60	178.0503	C9H8NO3[H-]	0.6	Hippuric Acid	Plasma	↑	4.73	32.13	2.08E-10	↑	6.32	2.75E-10	1	
						Liver	↑	7.09	12.42	4.89E-10	↑	4.50	4.91E-10		0.96
						Heart	↑	8.37	27.02	4.89E-10	↑	3.50	6.57E-08		0.98
						Kidney	↑	7.58	2.43	5.40E-10	↑	2.34	2.74E-09		0.73
6	1.82	417.1186	C21H21O9[H-]	0	Equol 7-Glucoronide	Plasma	↑	5.18	21.65	2.08E-10	↑	4.24	8.55E-05	1	
						Liver	↑	10.65	11.54	4.89E-10	↑	79.24	4.89E-10		0.88
						Heart	↑	8.14	34.49	4.89E-10	↑	214.44	4.89E-10		0.92
						Kidney	↑	7.89	5.04	4.89E-10	↑	70.97	4.89E-10		0.81
7	1.59	188.9862	C6H5O5S[H-]	2.1	Pyrocatechol Sulfate	Plasma	↑	2.56	33.91	2.08E-10	↑	7.14	2.08E-10	1	
						Liver	↑	6.21	39.39	4.89E-10	↑	7.62	4.89E-10		0.87
						Heart	↑	6.15	55.45	4.89E-10	↑	6.40	4.89E-10		0.88
						Kidney	↑	6.07	5.84	4.89E-10	↑	5.37	4.89E-10		0.75
8	1.67	283.0822	C13H15O7[H-]	1.4	P-Cresyl Glucuronide	Plasma	↑	2.28	16.80	2.14E-10	↑	21.57	2.83E-10	1	
						Liver	↑	2.35	4.60	5.27E-10	↑	10.71	3.68E-09		0.81
						Heart	↑	4.03	28.26	4.90E-10	↑	25.45	4.97E-10		0.99
						Kidney	↑	3.54	2.87	6.27E-08	↑	14.61	4.94E-10		0.85

^a^one plasma CKD outlier with a value of 14351% of control for p-cresyl sulfate was removed in the correlation analysis.
