# Supplementary material for: Untargeted plasma and tissue metabolomics in rats with chronic kidney disease given AST-120
Source: Sci Rep. 2016 Mar 2;6:22526. doi: 10.1038/srep22526 (PMC4773761; doi:10.1038/srep22526)
Supplement: Supplementary Information [file srep22526-s1.pdf]

Untargeted plasma and tissue metabolomics in rats with chronic kidney disease given AST-120.

Thomas J Velenosi[1], Anzel Hennop[1], David A Feere[1], Alvin Tieu[1], Andrew S Kucey[1], Polydoros Kyriacou[2], Laura E McCuaig[1], Stephanie E Nevison[1], Michael A Kerr[2], Bradley L Urquhart[1,3,4,*]

Supplemental Figure:

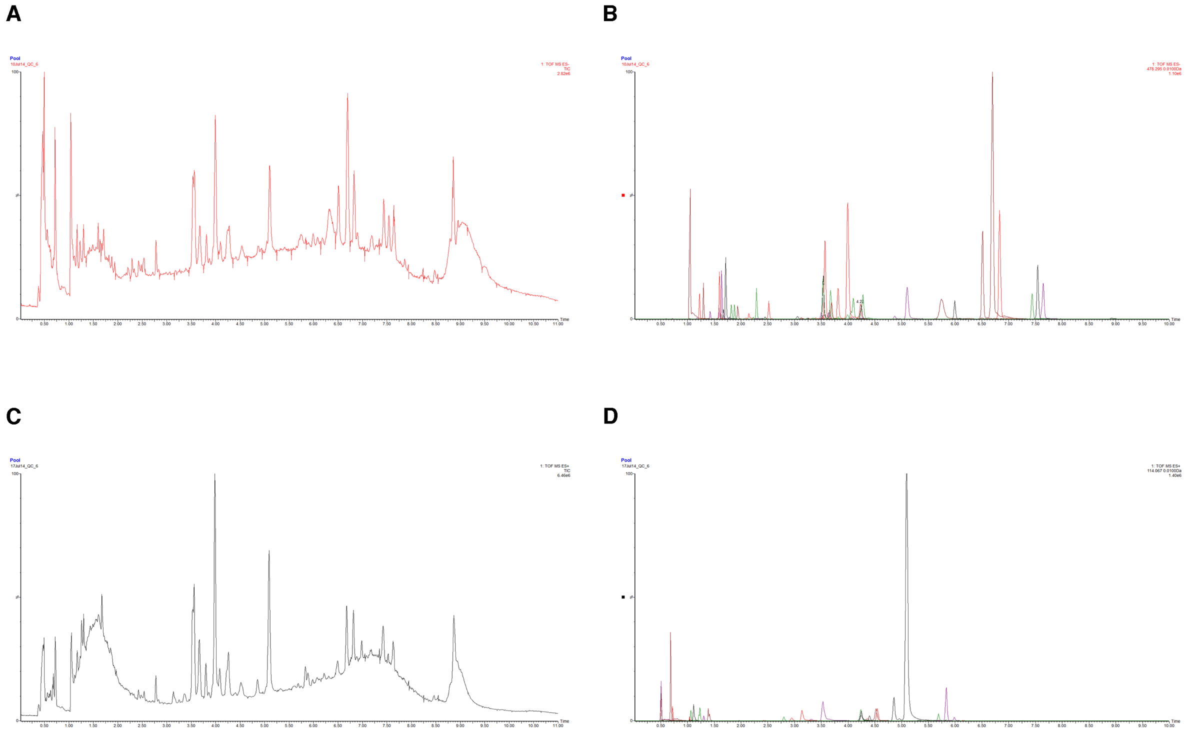

Supplemental Figure S1. Representative chromatograms of total ionic current (TIC) and extracted ions from a pooled rat kidney sample. Negative electrospray ionization mode TIC (A) and overlaid extracted ion chromatograms (B) of metabolites in Table 2 and Supplementary Table S5 from a single injection of pooled rat kidney sample. Positive electrospray ionization mode TIC (C) and overlaid extracted ion chromatograms (D) are also presented for a single injection.

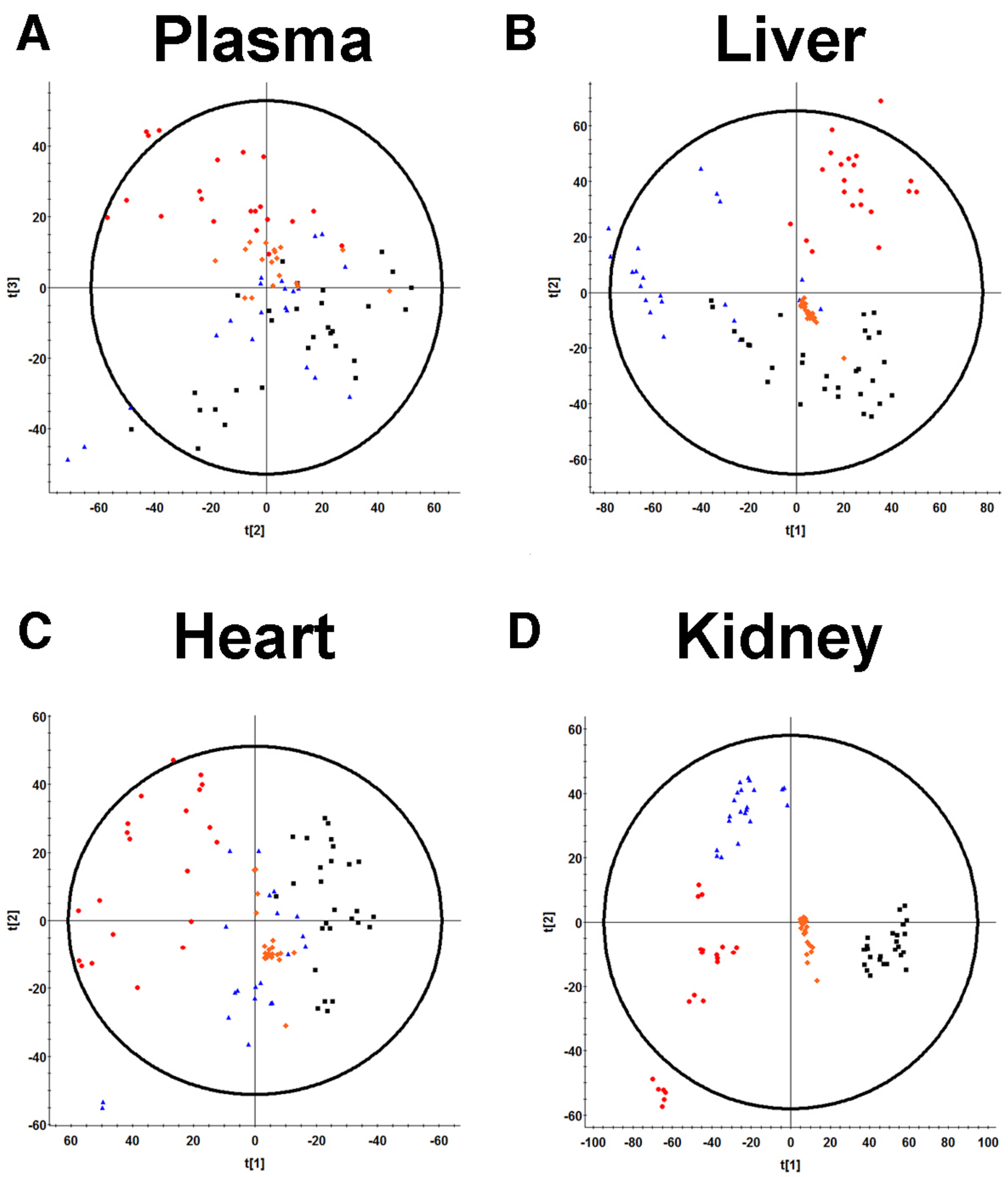

Supplemental Figure S2. Principle component analysis with pooled injections in negative ESI mode. Control (■), CKD (●), CKD+AST-120 (▲) and pooled (◆) negative ESI mode principle component analysis of plasma (A), liver (B), heart (C) and kidney (D) tissue in rats. Triplicate injections are shown.

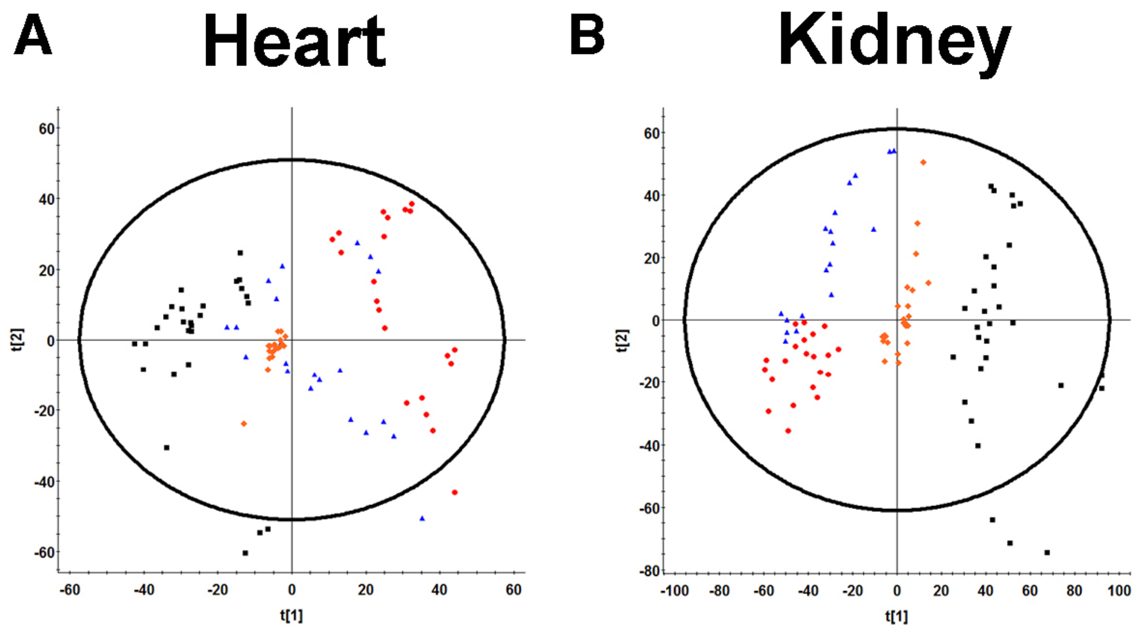

Supplemental Figure S3. Principle component analysis with pooled injections in positive ESI mode. Control (■), CKD (●), CKD+AST-120 (▲) and pooled (◆) negative ESI mode principle component analysis of heart (A) and kidney (B) tissue in rats. Triplicate injections are shown.

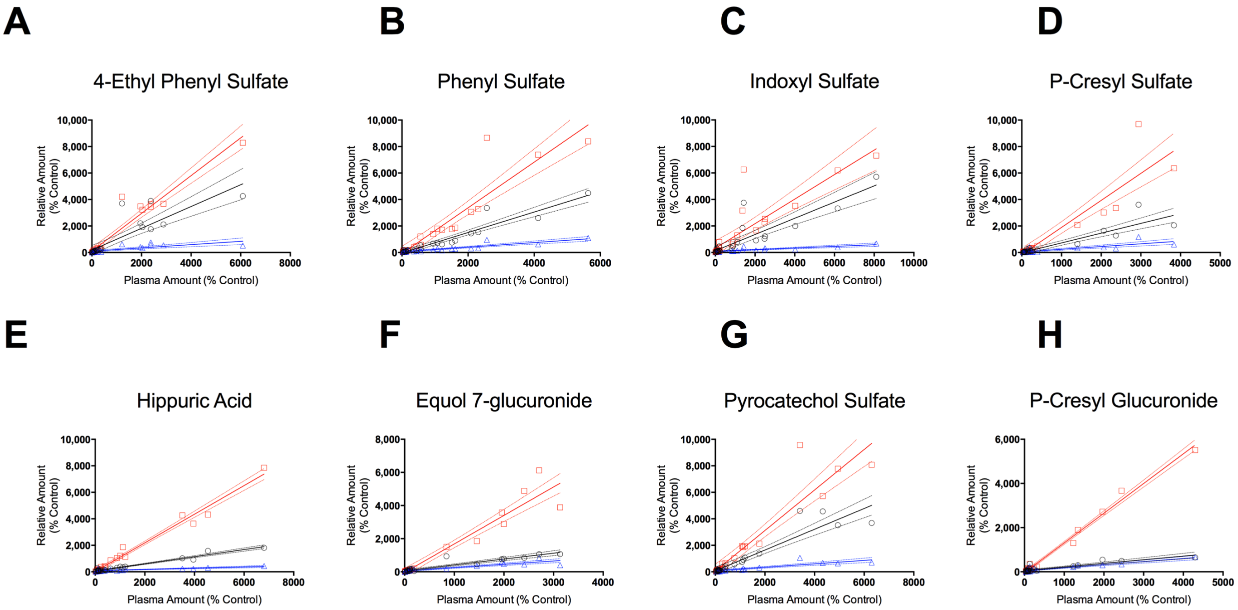

Supplemental Figure S4. Correlations between plasma and tissues for 8 gut-derived uremic toxins. Plasma levels of 4-ethylphenyl sulfate (A), phenyl sulfate (B), indoxyl sulfate (C), p-cresyl sulfate (D), hippuric acid (E), equol 7-glucuronide (F), pyrocatechol suflate (G) and p-cresyl glucuronide (H) were correlated to liver (○), heart (□) and kidney (△) levels for control, CKD and CKD+AST-120 groups. Linear regressions (solid lines) are presented with 95% confidence intervals (dashed lines).

Supplemental Table S5: Summary of metabolites altered in plasma, liver, heart and kidney tissue in rats with CKD compared to control and CKD+AST-120.

Ion	t_R (min)	Mass (m/z)	Empirical Formula	Mass Error (ppm)	Identity	Tissue	Change compared to Control				Change compared to CKD+AST-120			Metabolite ID Level
							Direction	S-plot VIP	Fold	P-value	Direction	Fold	P-value	
**9**	1.30	203.0818	C11H11N2O2[H-]	1.5	L-Tryptophan	Plasma	↓	2.86	0.34	2.08E-10	↓	0.68	5.65E-03	1
						Liver	↓	4.62	0.82	7.46E-06	↓	0.91	*1.13E-01*	
						Heart	↓	3.72	0.72	4.89E-10	↓	0.81	2.83E-08	
						Kidney	↓	6.49	0.41	4.89E-10	↓	0.70	5.80E-04	
**10**	2.15	514.2833	C26H44NO7S[H-]	1.6	Taurocholic acid	Plasma	↑	1.57	3.88	2.14E-05	↑	1.02	*1.00E+00*	2
						Liver	↑	10.38	1.68	2.79E-02	↑	1.40	*2.52E-01*	
						Heart	↑	0.80	1.42	*8.94E-02*	↓	0.81	*3.23E-01*	
						Kidney	↓	1.00	0.34	3.45E-03	↓	0.68	*8.64E-01*	
**11**	2.21	321.0443	C15H13O6S[H-]	-3.1	Equol 4-Sulfate	Plasma	NA				NA			2
						Liver	↑	10.38	10.38	4.89E-10	↑	160.97	4.89E-10	
						Heart	↑	0.04	1.15	*8.36E-01*	↑	28.71	4.90E-07	
						Kidney	↓	1.99	0.30	4.61E-09	↑	35.87	4.73E-02	
**12**	1.42	217.0176	C8H9O5S[H-]	2.3	Tyrosol 4-Sulfate	Plasma	NA				NA			2
						Liver	↑	6.39	129.42	4.99E-10	↑	67.42	7.62E-10	
						Heart	↑	4.85	291.44	4.98E-10	↑	55.28	7.88E-10	
						Kidney	↑	5.40	28.42	4.89E-10	↑	38.61	4.90E-10	
**13**	2.42	498.2896	C26H44NO6S[H-]	-1.4	Taurodeoxy(cheno)cholic acid	Plasma	NA				NA			3
						Liver	↓	14.06	0.60	6.77E-03	↓	0.59	1.17E-02	
						Heart	↓	0.88	0.63	*2.99E-01*	↓	0.27	9.09E-10	
						Kidney	↓	1.18	0.28	6.68E-03	↓	0.44	*4.40E-01*	
**14**	6.67	303.2335	C20H31O2[H-]	-3.6	Arachidonic Acid	Plasma	NA				NA			2
						Liver	↓	11.59	0.89	*7.64E-01*	↑	1.10	*9.20E-01*	
						Heart	↑	7.10	1.05	*4.28E-01*	↑	1.05	*5.32E-01*	
						Kidney	↓	1.18	0.85	1.00E-04	↓	0.87	4.81E-03	
**15**	6.80	279.2332	C18H31O2[H-]	-2.9	Linoleic Acid	Plasma	NA				NA			2
						Liver	↓	9.01	0.95	*6.72E-01*	↓	0.87	2.81E-02	
						Heart	↑	7.66	1.00	*1.00E+00*	↑	1.04	*8.62E-01*	
						Kidney	↓	10.07	0.66	7.63E-08	↓	0.78	1.57E-02	
**16**	7.40	255.2334	C16H31O2[H-]	0	Palmitic Acid	Plasma	↓	0.90	0.97	*9.98E-01*	↑	1.00	*1.00E+00*	1
						Liver	↓	11.99	0.78	*8.98E-02*	↓	0.55	1.37E-08	
						Heart	↑	0.53	1.16	6.33E-03	↑	1.02	*9.79E-01*	
						Kidney	↓	4.74	0.66	2.66E-04	↓	0.79	*2.07E-01*	
**17**	7.61	281.2491	C18H33O2[H-]	-3.5	Oleic Acid	Plasma	NA				NA			1
						Liver	↓	10.68	0.85	*4.12E-01*	↓	0.64	1.33E-04	
						Heart	↓	0.36	0.83	*8.06E-01*	↓	0.89	*9.59E-01*	
						Kidney	↓	3.63	0.81	*1.56E-01*	↓	0.73	1.36E-02	
**18**	5.97	301.2178	C20H29O2[H-]	-3.3	Eicosapentaenoic Acid	Plasma	NA				NA			2
						Liver	↓	9.53	0.84	*2.29E-01*	↓	0.67	1.05E-04	
						Heart	↑	5.59	2.00	7.96E-08	↑	1.89	2.70E-06	
						Kidney	↓	5.05	0.51	5.69E-10	↓	0.71	1.90E-02	
**19**	6.51	327.2335	C22H31O2[H-]	3.4	Docosahexaenoic Acid	Plasma	NA				NA			2
						Liver	↓	7.29	0.98	*9.91E-01*	↓	0.78	1.02E-02	
						Heart	↑	2.67	1.16	4.03E-02	↑	1.09	*4.72E-01*	
						Kidney	↓	14.17	0.38	4.89E-10	↓	0.75	2.83E-02	

**20**	1.23	218.1037	C9H16NO5[H-]	4.1	D-pantothenic Acid	Plasma	NA				NA			2
						Liver	↑	2.92	1.01	*9.99E-01*	↑	1.09	*8.18E-01*	
	1.23	220.1194	C9H18NO5[H+]	4.1		Heart	↓	11.72	0.86	*9.58E-01*	↓	0.47	1.49E-02	
						Kidney	↓	4.10	0.67	5.19E-10	↑	1.65	3.56E-07	
**21**	1.88	297.0975	C14H17O7[H-]	-0.3	Phenylethanol Glucoronide	Plasma	NA				NA			2
						Liver	↑	4.61	7.91	4.89E-10	↑	27.74	4.89E-10	
						Heart	↑	6.14	54.66	4.89E-10	↑	46.89	4.89E-10	
						Kidney	↑	7.73	6.42	4.89E-10	↑	42.09	4.89E-10	
**22**	0.70	306.0771	C10H16N3O6S[H-]	3.6	Glutathione	Plasma	NA				NA			1
						Liver	↓	5.04	0.84	*7.18E-01*	↑	1.34	*5.63E-01*	
	0.72	308.0923	C10H18N3O6S[H+]	2.3		Heart	↑	7.67	1.70	5.37E-05	↓	0.98	*9.94E-01*	
						Kidney	↓	0.57	0.07	5.26E-10	↓	0.49	*9.37E-01*	
**23**	7.54	331.2647	C22H35O2[H-]	3.0	Adrenic Acid	Plasma	NA				NA			2
						Liver	↓	3.43	0.87	*7.59E-01*	↓	0.56	5.67E-05	
						Heart	↑	3.96	1.62	8.96E-07	↑	1.30	1.09E-02	
						Kidney	↑	10.13	2.57	4.89E-10	↓	0.78	2.79E-06	
**24**	1.63	245.0124	C9H11O7S (-H2O)[H-]	1.6	3-Methoxy-4-Hydroxyphenylglycol sulfate	Plasma	NA				NA			2
						Liver	↑	3.10	18.14	4.89E-10	↑	10.04	4.89E-10	
						Heart	↑	3.50	106.17	4.89E-10	↑	10.22	4.89E-10	
						Kidney	↑	4.70	7.98	4.89E-10	↑	6.94	4.89E-10	
**25**	3.98	540.3313	C25H51NO9P[CHOOH-]	2.2	LysoPC(16:0)	Plasma	↑	1.42	2.38	1.30E-04	↑	1.26	*4.71E-01*	3
						Liver	↑	5.68	1.01	*9.92E-01*	↑	1.01	*9.83E-01*	
	3.99	496.3407	C24H51NO7P[H+]	0.8		Heart	↓	10.10	0.91	*6.26E-02*	↓	0.99	*9.98E-01*	
						Kidney	↑	1.90	1.70	4.89E-10	↑	1.05	*4.07E-01*	
**26**	3.67	564.3316	C26H49NO7P[CHOOH-]	2.7	LysoPC(18:2)	Plasma		NA				NA		3
						Liver	↑	2.95	1.02	*9.80E-01*	↓	0.94	*5.02E-01*	
	3.67	520.3412	C26H51NO7P[H+]	1.7		Heart	↓	9.70	0.66	8.72E-10	↓	0.83	4.32E-02	
						Kidney	↓	2.27	0.83	1.69E-02	↑	1.09	*6.92E-01*	
**27**	3.63	476.2791	C23H43NO7P[H-]	2.9	LysoPE (18:2)	Plasma	NA				NA			3
						Liver	↓	3.22	0.75	1.12E-03	↓	0.63	3.60E-08	
	3.67	520.3412	C26H51NO7P[H+]	1.7		Heart	↓	6.94	0.60	3.25E-07	↓	0.77	*6.21E-02*	
						Kidney	↓	3.27	0.38	4.46E-09	↓	0.97	*9.99E-01*	
**28**	4.25	436.2837	C21H43NO6P[H-]	2.1	PE(P-16:0/0:0)	Plasma	NA				NA			3
						Liver	↓	0.38	0.95	*9.93E-01*	↓	0.60	1.06E-02	
						Heart	↓	5.60	0.76	1.59E-05	↓	0.83	2.78E-02	
						Kidney	↑	5.50	2.54	4.89E-10	↑	1.24	5.99E-03	
**29**	5.66	619.2898	C29H48O12P[H-]	2.4	(PI)20:4)	Plasma	↑		30.87	2.11E-10	↑	3.72	2.93E-07	3
						Liver	↑	1.45	1.25	2.80E-03	↓	0.86	4.56E-02	
						Heart	↓	4.90	0.89	*2.22E-01*	↓	0.94	*8.08E-01*	
						Kidney	↓	7.50	0.37	4.89E-10	↓	0.67	8.60E-04	
**30**	3.57	588.3313	C29H51NO9P[HCOOH-]	2.0	LysoPC(20:4)	Plasma	NA				NA			3
						Liver	↑	5.39	1.07	2.52E-04	↑	1.07	1.41E-03	
	3.57	544.3408	C28H51NO7P[H+]	0.9		Heart	↑	1.40	1.10	*6.25E-02*	↑	1.21	7.91E-05	
						Kidney	↑	9.70	1.69	4.89E-10	↓	0.99	*9.99E-01*	
**31**	4.10	566.3472	C27H53NO9P[HCOOH-]	2.5	LysoPC(18:1)	Plasma	↑		2.71	9.89E-05	↑	1.83	1.74E-02	3
						Liver	↑	3.41	1.14	*3.05E-01*	↑	1.27	2.54E-02	
	4.08	522.3566	C26H53NO7P[H+]	1.1		Heart	↑	0.58	1.17	*6.80E-02*	↑	1.20	4.71E-02	
						Kidney	↑	6.66	2.46	4.89E-10	↑	1.04	*8.59E-01*	
**32**	5.11	508.3415	C25H51NO7P[H-]	2.4	LysoPC(17:0) or LysoPE(20:0)	Plasma	NA				NA			3
						Liver	↑	1.45	1.15	1.47E-02	↑	1.04	*8.84E-01*	
						Heart	↓	3.72	0.88	*8.49E-01*	↓	0.74	*2.32E-01*	
						Kidney	↑	5.18	1.44	4.89E-10	↑	1.24	1.66E-08	

**33**	3.54	500.2787	C25H43NO7P[H-]	2.0	LysoPE(20:4)	Plasma	NA				NA			3
						Liver	↓	6.62	0.92	*1.79E-01*	↓	0.86	1.54E-03	
						Heart	↑	2.34	1.07	*2.17E-01*	↑	1.14	7.61E-03	
						Kidney	↓	9.65	0.45	4.89E-10	↓	0.84	2.94E-03	
**34**	1.05	267.0739	C10H11N4O5[H-]	3.7	Inosine	Plasma	NA				NA			1
						Liver	↓	6.05	0.87	*8.33E-01*	↑	1.14	*9.28E-01*	
						Heart	↑	2.59	1.07	*9.57E-01*	↑	1.16	*7.75E-01*	
						Kidney	↓	9.24	0.24	1.10E-09	↑	3.86	*4.06E-01*	
**35**	4.23	478.2945	C23H45NO7P[H-]	2.3	LysoPE(18:1)	Plasma	NA				NA			3
						Liver	↓	2.14	0.87	*1.88E-01*	↓	0.92	*7.38E-01*	
						Heart	↓	2.79	0.85	*4.05E-01*	↑	1.03	*9.94E-01*	
						Kidney	↓	5.36	0.57	4.89E-10	↓	0.99	*9.98E-01*	
**36**	2.42	464.3021	C26H42NO6[H-]	1.9	Glycocholic Acid	Plasma	NA				NA			2
						Liver	↑	2.73	1.29	*5.53E-02*	↑	2.98	1.99E-09	
						Heart	↑	1.04	1.36	*8.91E-02*	↑	2.34	4.05E-05	
						Kidney	↓	0.85	0.23	4.66E-09	↑	1.38	*9.52E-01*	
**37**	5.69	403.2853	C25H39O4[H+]	1.2	Monoacylglyceride(22:6)	Heart	↑	26.34	3.88	3.56E-10	↑	1.64	3.45E-09	3
						Kidney	↑	1.81	1.43	*1.68E-01*	↓	0.49	1.39E-08	
**38**	5.83	379.2852	C23H39O4[H+]	1.1	1-Arachidonyl Glycerol	Heart	↑	14.40	2.85	3.56E-10	↑	1.13	*1.51E-01*	3
						Kidney	↑	9.32	3.36	2.62E-08	↑	1.08	*9.30E-01*	
**39**	4.25	572.3721	C30H55NO7P[H+]	0.9	LysoPC(22:4)	Heart	↑	11.33	1.87	3.57E-10	↑	1.47	1.23E-06	3
						Kidney	↑	9.13	12.69	4.89E-10	↑	1.35	1.03E-07	
**40**	0.68	204.1242	C9H18NO4[H+]	2.9	Acetyl-Carnitine	Heart	↓	41.98	0.51	1.17E-07	↓	0.91	*9.27E-01*	2
						Kidney	↓	1.22	0.96	*9.24E-01*	↓	0.92	*6.27E-01*	
**41**	1.04	248.1504	C11H22NO5[H+]	2.4	Hydroxybutyrylcarnitine	Heart	↓	12.73	0.24	3.57E-10	↓	0.28	3.34E-08	2
						Kidney	↓	1.42	0.56	3.60E-02	↓	0.65	*3.15E-01*	
**42**	1.23	232.1557	C11H22NO4[H+]	3.4	Butyryl-L-Carnitine	Heart	↓	27.04	0.23	3.56E-10	↓	0.39	2.33E-05	2
						Kidney	↓	3.04	0.67	4.62E-08	↑	1.04	*9.71E-01*	
**43**	3.51	426.3587	C25H48NO4[H+]	0.9	Oleolyl, 11-Octadecenyl,<br>Elaidic, or Vaccenyl Carnitine	Heart	↓	27.89	0.38	1.37E-09	↓	0.50	6.91E-04	3
						Kidney	↓	3.11	0.72	1.69E-04	↓	0.61	6.13E-09	
**44**	4.20	454.39	C27H52NO4[H+]	0.9	11-cis-Icoseneoylcarnitine	Heart	↓	22.32	0.27	2.42E-09	↓	0.33	2.37E-05	2
						Kidney	↓	0.42	0.83	*7.62E-02*	↓	0.66	2.01E-06	
**45**	0.50	162.1139	C7H16NO3[H+]	5.6	L-Carnitine	Heart	↓	21.64	0.39	3.56E-10	↓	0.55	6.22E-10	2
						Kidney	↓	9.02	0.29	3.42E-03	↑	1.06	*1.00E+00*	
**46**	2.95	442.3536	C25H48NO5[H+]	0.9	3-Hydroxyoctadecenoylcarnitine	Heart	↓	21.16	0.23	3.56E-10	↓	0.46	2.79E-04	2
						Kidney	↑	3.23	2.67	1.21E-06	↓	0.49	4.91E-10	
**47**	2.80	416.3378	C23H46NO5[H+]	0.5	Hydroxyhexadecanoylcarnitine	Heart	↓	18.87	0.25	3.56E-10	↓	0.52	4.07E-03	2
						Kidney	↑	2.22	1.61	7.56E-03	↓	0.50	5.12E-10	
**48**	3.30	444.3691	C25H50NO5[H+]	0.5	12-Hydroxy-12-Octadecenoylcarnitine	Heart	↓	17.47	0.23	3.56E-10	↓	0.43	1.72E-03	2
						Kidney	↓	0.36	0.66	1.07E-05	↓	0.57	5.71E-09	
**49**	5.10	524.3719	C26H55NO7P[H+]	0.6	LysoPC(18:0)	Heart	↓	2.03	0.99	*9.99E-01*	↓	0.91	*3.29E-01*	3
						Kidney	↑	24.72	1.56	1.45E-09	↑	1.29	5.22E-04	
**50**	4.52	508.3771	C26H55NO6P[H+]	0.8	1-(octadecenyl)-sn-Glycero-3-Phosphocholine	Heart	↑	2.10	1.14	*2.25E-01*	↑	1.21	*6.43E-02*	3
						Kidney	↑	10.06	7.50	4.89E-10	↓	0.92	*1.79E-01*	
**51**	4.55	548.3721	C28H55NO7P[H+]	0.4	LysoPC(20:2)	Heart	↓	1.06	0.99	*9.97E-01*	↑	1.23	2.79E-02	3
						Kidney	↑	9.81	4.90	4.89E-10	↑	1.18	3.12E-03	
**52**	4.24	482.3613	C24H53NO6P[H+]	0.6	3-Hexadecyl-sn-Glycero-1-Phosphocholine	Heart	↓	2.04	0.94	*8.23E-01*	↓	1.00	*1.00E+00*	3
						Kidney	↑	9.07	4.86	4.89E-10	↓	1.00	*1.00E+00*	
**53**	0.51	258.1115	C8H21NO6P[H+]	3.5	Glycerophosphocholine	Heart	↑	9.96	1.39	*8.41E-01*	↑	0.70	*6.64E-01*	2
						Kidney	↑	8.77	1.33	*8.33E-01*	↓	0.71	*5.62E-01*	

**54**	1.12	257.1149	C11H16N2O5	4.7	1-beta-d-Ribofuranosyl-1,4-dihydronicotinamide	Heart	↓	3.65	0.30	3.01E-08	↓	0.55	3.56E-10	2
						Kidney	↓	10.00	0.07	4.89E-10	↓	1.06	*1.00E+00*	
**55**	1.40	246.1712	C12H24NO4[H+]	2.8	2-methylbutyroylcarnitine or pivaloycarnitine	Heart	↓	9.87	0.26	3.56E-10	↓	0.40	3.56E-10	3
						Kidney	↓	8.48	0.07	4.89E-10	↓	0.64	*8.54E-01*	
**56**	3.12	424.3431	C25H46NO4[H+]	0.9	Linoleyl or Linoelaidyl Carnitine	Heart	↓	17.44	0.47	6.26E-10	↓	0.67	1.53E-02	3
						Kidney	↓	7.06	0.33	4.89E-10	↓	0.63	8.63E-04	
**57**	1.06	262.1661	C12H24O5N1[H+]	2.7	3-Hydroxyisovalerylcarnitine	Heart	↓	6.92	0.42	3.56E-10	↓	0.51	7.99E-08	2
						Kidney	↓	7.61	0.13	4.89E-10	↓	0.70	*4.90E-01*	

Supplementary Table S6. Metabolite pathway analysis using MetaboAnalyst 3.0

Number	Pathway	Tissue	Total	Hits	Raw p	-LOG(p)	Holm Adjust	FDR	Impact
1	**Phenylalanine metabolism**	Plasma	45	1	9.27E-10	20.799	6.49E-09	6.49E-09	0.0315
		Liver	45	1	8.50E-11	23.189	1.10E-09	2.89E-10	0.0315
		Heart	45	1	2.52E-08	17.495	3.28E-07	1.26E-07	0.0315
		Kidney	45	1	2.01E-07	15.419	2.41E-06	6.44E-07	0.0315
2	**Primary bile acid biosynthesis**	Plasma	47	1	2.77E-06	12.795	1.66E-05	6.47E-06	0.00846
		Liver	47	2	0.024685	3.7015	0.14811	0.034971	0.01692
		Heart	47	2	0.017285	4.0579	0.121	0.028809	0.01692
		Kidney	47	2	1.12E-05	11.399	7.84E-05	1.79E-05	0.01692
3	**Taurine and hypotaurine metabolism**	Plasma	20	1	2.77E-06	12.795	1.66E-05	6.47E-06	0
		Liver	20	1	1.23E-06	13.611	1.47E-05	3.48E-06	0
		Heart	20	1	0.01284	4.3552	0.11015	0.024076	0
		Kidney	20	1	0.010651	4.5421	0.021301	0.011361	0
4	**Glycerophospholipid metabolism**	Plasma	39	1	0.00018895	8.574	0.00075579	0.00033066	0.00317
		Liver	39	2	0.85934	0.15159	1	0.85934	0.1307
		Heart	39	2	0.012239	4.4031	0.11015	0.024076	0.1307
		Kidney	39	3	2.70E-07	15.124	2.97E-06	7.20E-07	0.14055
5	**Fatty acid metabolism**	Plasma	50	1	0.25548	1.3646	0.76645	0.25548	0.02959
		Liver	50	1	0.002964	5.8212	0.023712	0.0045808	0.02959
		Heart	50	1	0.0045676	5.3888	0.050243	0.011419	0.02959
		Kidney	50	1	0.0010536	6.8555	0.005268	0.0012041	0.02959
6	**Fatty acid biosynthesis**	Plasma	49	1	0.25548	1.3646	0.76645	0.25548	0
		Liver	49	2	0.0017773	6.3327	0.015996	0.0033571	0
		Heart	49	2	0.11603	2.1539	0.69617	0.17404	0
		Kidney	49	1	0.0010536	6.8555	0.005268	0.0012041	0
7	**Fatty acid elongation in mitochondria**	Plasma	27	1	0.25548	1.3646	0.76645	0.25548	0
		Liver	27	1	0.002964	5.8212	0.023712	0.0045808	0
		Heart	27	1	0.0045676	5.3888	0.050243	0.011419	0
		Kidney	27	1	0.0010536	6.8555	0.005268	0.0012041	0
8	**Pantothenate and CoA biosynthesis**	Plasma	NA						
		Liver	27	1	5.37E-11	23.647	8.06E-10	2.28E-10	0.18014
		Heart	27	1	0.50957	0.6742	1	0.58796	0.18014
		Kidney	27	1	8.11E-08	16.328	1.14E-06	3.24E-07	0.18014
9	**beta-Alanine metabolism**	Plasma	NA						
		Liver	28	1	5.37E-11	23.647	8.06E-10	2.28E-10	0
		Heart	28	1	0.50957	0.6742	1	0.58796	0
		Kidney	28	1	8.11E-08	16.328	1.14E-06	3.24E-07	0
10	**Glutathione metabolism**	Plasma							
		Liver	38	1	6.60E-06	11.928	7.26E-05	1.40E-05	0.23743
		Heart	38	1	1.30E-08	18.156	1.95E-07	9.77E-08	0.23743
		Kidney	38	1	4.98E-07	14.512	4.98E-06	9.97E-07	0.23743
11	**Cysteine and methionine metabolism**	Plasma	NA						
		Liver	56	1	6.60E-06	11.928	7.26E-05	1.40E-05	0.00735
		Heart	56	1	1.30E-08	18.156	1.95E-07	9.77E-08	0.00735
		Kidney	56	1	4.98E-07	14.512	4.98E-06	9.97E-07	0.00735
12	**Arachidonic acid metabolism**	Plasma	NA						
		Liver	62	1	0.027914	3.5786	0.14811	0.036503	0.21669
		Heart	62	1	0.23483	1.4489	1	0.32022	0.21669
		Kidney	62	1	0.00040259	7.8176	0.0024155	0.00058558	0.21669
13	**Linoleic acid metabolism**	Plasma	NA						
		Liver	15	1	0.035123	3.3489	0.14811	0.042649	0.65625
		Heart	15	1	0.97705	0.023214	1	0.97705	0.65625
		Kidney	15	1	3.04E-06	12.703	2.43E-05	5.41E-06	0.65625
14	**Purine metabolism**	Plasma	NA						
		Liver	92	1	0.43236	0.8385	1	0.49001	0.00425
		Heart	92	1	0.61199	0.49104	1	0.6557	0.00425
		Kidney	92	1	1.17E-09	20.566	1.76E-08	9.36E-09	0.00425
15	**Glycosylphosphatidylinositol(GPI) -anchor biosynthesis**	Plasma							
		Liver	14	1	0.66349	0.41024	1	0.70496	0.0439
		Heart	14	1	0.0002639	8.2399	0.0031669	0.00098964	0.0439
		Kidney	14	1	2.63E-11	24.361	4.21E-10	4.21E-10	0.0439
16	**Ether lipid metabolism**	Kidney	23	1	0.37906	0.97007	0.37906	0.37906	0
17	**Starch and sucrose metabolism**	Liver	50	1	5.46E-18	39.749	9.28E-17	4.64E-17	0.01265
18	**Pentose and glucuronate interconversions**	Liver	53	1	5.46E-18	39.749	9.28E-17	4.64E-17	0.009

Note: Total is the total number of compounds in the pathway; Hits are the number of compounds matched in that pathway; the Raw p is the original p value calculated from the enrichment analysis; the Holm p is the p value adjusted by Holm-Bonferroni method; the FDR p is the p value adjusted using False Discovery Rate; the Impact is the pathway impact value calculated from pathway topology analysis.
